# Ulceration of Cornea Cured by Lac Felinum

**Published:** 1883-11

**Authors:** E. W. Berridge

**Affiliations:** London


### ULCERATION OF CORNEA CURED BY LAC FELINUM.

E. W. Berridge, M. D., London.

In the Homœopathic Physician for June I published two ophthalmic cases treated by this remedy. To the latter of these cases I now add the following report:

"May 4th, 1882.—Reports that the letters do not run together, though sight is not good; the pains and the illusions have been only very slight since former report, appearing only when tired, and not at all now for a long time." The symptoms of both these cases, therefore, verify Dr. Swan's proving, as does also the following case, which is still more remarkable.

Miss F. M., æt. 21. January 26th, 1878.—Several years ago had ulcer on right cornea; some liquid was put into the eye to dilate the pupil and a setor inserted in temple, but without benefit; then she went to a convalescent home and came back well, but on catching cold her right eye became inflamed. For this attack I prescribed and cured her (I cannot find my notes of this attack). The eye remained well until the end of last September, when the right eye became red with photophobia and pain. She then consulted a pseudo-homœopath, remaining under his care for six or eight weeks. The eye improved, but subsequently became much worse. She then went to a pseudo-homœopathic institution, where she was treated for

---

*The eye cannot be cured without the whole head,
Nor the head without the whole body,
Nor the whole body without the spirit.

two months, something being put into the eye. She was dismissed as cured in three weeks, and was told that she would never get bad again; however, in three days she became worse than ever.

*Present state.*—Whitish ulcer in right cornea over pupil; right eyelids red and swollen; right eye waters. *Darting pain going backward in centre of right eye,* worse at night. This is the latest symptom. If she lies on left side, right eye feels as if it were moving about and were too heavy, with great pain. With the right eye everything seems in a white fog. Photophobia of right eye; always feels tired. *Lac felinum*$^{40m}$ (Fincke), one dose.

Feb. 1st.—Lids less red and swollen. Lachrymation almost gone. Darting pain much less. Feeling of moving about of eye better. Much less photophobia. Since January 29th, left eye feels hot and adheres in morning. Other symptoms are unchanged.

Feb. 9th.—Ulcer appears to be healing. Lids much better. No lachrymation. No return of darting pain till last night. The feeling of moving about and heaviness with pain has gone for a week. Photophobia much better. Other symptoms unchanged. For last week white spots on outer edge of left cornea, with red vessels running up to it from conjunctiva, and shooting pains from the spot to occiput. Says the right eye originally got bad in this way.

Feb. 16th.—No return of lachrymation. No more darting in right eye. Less photophobia. No pain in left eye for a week. Both eyes look better. On left eye the spot is less and has very few vessels. On the left eye the spot is thinner at circumference. No other change since last report.

Feb. 23d.—Ulcer on right eye better. Lids quite well. Photophobia much better. Heat and adhesion of left eye better.

March 2d.—The symptoms of left cornea, which appeared on February 9th, have returned. There is an ulcer on junction of left cornea and conjunctiva, with red vessels from outer conjunctiva running up to it, and a little shooting pain. Lachrymation of right eye returned for last week. Hot pain in right eye. Darting in right eye returned yesterday. Less heat and adhesion of left eye.

March 9th.—Has felt better for the last two days. Lachrymation gone. Darting in right eye not returned. Photophobia gone. All the week, with the exception of the last two days, left eye has felt hot and as if something were in it; it adheres on waking in morning. Darting in left eye gone for four days, and the eye looks better.

March 16th.—Has felt better all the week. Spot on right eye less; that on left eye nearly healed. Still feels tired. White fog less. No other symptoms.

April 13th.—Has steadily improved all the time. Has had no more pain in either eye. White fog better for about a month. Still feels tired.

April 27th.—On 24th pain in lower border of right orbit, as if sore, with tenderness there on touch, and shooting in right eye as before; no other pain since last report. Ulcer on right eye better. Much less white fog; can read better.

May 11th.—No return of pain. White fog nearly gone. White spot less. Still tired. No other symptoms.

May 25th.—Has had no more pain, except on 19th, 20th, and 21st, when she had the old darting pain in right eye with heat therein; also swelling of right cheek, nausea, light-headed feeling, heaviness of occiput, very cold and very weak. She had these symptoms when the eye first got bad in this attack, and also in the attack seven years ago. Spot smaller; sight better.

June 8th.—On the 4th right eye was painful, as if she had a cold in it; it was hot, red, and adhered next morning on waking. No other pain since last report. White fog increased this week, and white spot on right eye remains unchanged. No other symptoms.

June 22d.—No more pain in eyes. Sore pain around right eye for two days.

Aug. 9th.—For the last three weeks has been at a convalescent home at the seaside. The allopathic physician there said on the first interview that he did not expect to see it so well, and that she ought to be thankful to be able to see at all; when he saw her again, just before she left, he said it was doing very well, but might get bad again in the winter, and that the spot would never go away. To-day the white spot is very small. Still some white fog before right eye. Has had no pain or other symptoms of eyes since last report, except a sty in right lower lid last week. Feels very well generally; no tiredness.

Aug. 31st.—About a week ago, feeling of dust in right eye for two days; no other pain since last visit. Still has the white fog, but much less than at first; has also white spot. No other symptoms.

Sept. 21st.—On the 15th right eye became inflamed, remaining so till 18th, with shooting backward where the white spot is. Left eye was also inflamed at the same time, but without any shooting; on 18th there appeared a white spot on inner segment of left cornea, lasting till 20th, with shooting backward there. Since 18th, sty on left upper lid. For last two weeks both eyes have ached by gaslight. For the last week, with the exception of to-day, right eye has watered and adhered on waking in morning. White spot and white mist unchanged. She says her eyes got bad just a year ago. *Lac felinum*$^{40m}$ (Fincke), one dose.

Sept. 28th.—Lachrymation of right eye less; has had very little adhesion of lids; no pain or redness of right eye. Both eyes ache by gaslight as before. For last week has had shootings backward in middle of left eye; now there is redness in lower inner segment of left eye, the vessels running up to a vesicle where conjunctiva joins cornea at lower inner segment (not *on* the cornea). White spot on right eye less; white mist unchanged.

Oct. 5th.—Up to October 1st there was lachrymation and adhesion of left eye, but not since. No return of symptoms of right eye. Left eye much inflamed on September 30th and October 1st, with the shooting as formerly. Eyes still ache by gaslight. White fog remains. White spot on right eye less. Now scarcely any redness of left eye and no vesicle.

Oct. 19th.—No more pain in eyes, except aching by gaslight. White fog still. A week ago had a bad sty on right upper lid, near inner canthus; it broke on 17th and again on 18th; it was hot, throbbing, and very much inflamed; since the sty formed, lachrymation of right eye; and since it broke, adhesion of right eye.

Nov. 2d.—On October 21st to 23d, left eye inflamed with burning shooting going to the left occiput, adhesion of lids on waking in morning, and eye full of yellow pus; after 23d no pain, redness better and gone by 26th. For two days sty forming on left lower lid. Still aching in eyes by gaslight. Spotless. White fog still. For a week second lower left molar has been painful, a sharp pain, worse at night, and the tooth is loose. It was bad before she first consulted me, when her right eye was bad.

Nov. 16th.—Sty disappeared without discharging. No pains for fourteen days, except aching by gaslight. White fog still. Tooth firm and painless for a week. White spot fainter. No other symptoms for fourteen days.

Dec. 7th.—No more pains or sties, except aching by gaslight. White fog better for a week, and could see very well. The tooth broke off when talking, three days ago.

Dec. 28th.—No return of pain till to-day, when there has been pain as from something in right eye, which watered. Sight better. White fog less.

March 1st, 1879.—No more pains; lachrymation only in very cold weather. White fog quite gone. Sight perfect. Spot much less.

April 5th.—Eye has felt *quite well* till about six days ago; since then right eye has been inflamed with shooting and heat in it, followed by shooting from right internal ear to occiput and vertex; to-day much less pain in head, none in eye. Eyes both well, except the spot, which is smaller. Two years ago had the same kind of pains in right head.

May 10th.—No more pain, except for three days a week ago, when she had hot pains in both eyes, with shooting from middle of eyes to occiput. For two days sty on left lower lid. Spot unchanged.

June 29th.—No return of pain in eyes, except one day about a week ago, when she had neuralgia in head, and then right eye ached with shooting backward, as before. Spot still on eye. Sight good. *Lac felinum*$^{40m}$ (Fincke), one dose.

July 26th.—Has been very well till last week, when right eye was very painful, as before on two or three occasions.

Sept. 27th.—On September 1st and 2d right eye was painful, watered, and lids adhered on waking; now for six days right eye has been inflamed, with the former shooting pains and adhesion of lids in morning. White spot still. White mist has returned. *Lac felinum*$^{cm}$ (Fincke), seven doses, one every morning.

March 16th, 1880.—Reports that eye got well in five days; white mist disappeared. On Decenber 12th sty on left upper lid toward inner canthus; it disappeared without discharging. Last week she had a bad cough, and eyes have ached, with the shooting through to occiput, as formerly, at times. *Lac felinum*$^{cm}$ (Fincke), for discharges for six days.

April 3d.—No pain in eyes since medicine was finished. White mist before right eye by gaslight.

Sept. 4th.—On April 29th and 30th had a return of shooting in right eye as before, but there was no mist and the film was less. For a week white mist before right eye every morning on rising till she goes out, at 8.30 A. M., and again in evening by gaslight. Once or twice shooting in right eye, and all the week feeling of dust in it. Says her eye always feels bad in September. *Lac felinum*$^{cm}$ (Fincke) every morning for seven days.

Oct. 2d.—No more pains till September 26th; since right eye has felt as from dust in it, and shooting in it as formerly; left eye has ached in the light. The mist has been coming on again this last week. *Lac felinum*$^{cm}$ (Fincke) every morning for fourteen days.

Oct. 16th.—The shooting was better the first week, but worse this week; the white mist, which had ceased, has returned this week.

Nov. 13th.—Has felt quite well for three weeks; no mist or pain.

May 26th, 1881.—Had two sties on right lower lid in March, otherwise eyes have felt quite well till 22d, when right eye became inflamed, getting worse every day since. Now right eye feels hot, with shooting going back through centre of eye to occupit; right face and right head very painful, better by lying on painful side; eye not affected in position; photophobia; inner surface of right lids red; film on corner rather increased. *Lac felinum*$^{cm}$ (Fincke) twice a day for six days.

April 21st, 1882.—Has twice had sty on right lower lid, which had healed without trouble. Has had no more trouble with eyes; no pain, no inflammation, no photophobia; no return of white mist. No film on left eye, and only a small, faint film on centre of right cornea.

Aug. 4th.—Last week, shooting returned in centre of right eye, but with only a little redness; otherwise has been well since last report. *Lac felinum*$^{cm}$ (Fincke) every morning for seven days.

Oct. 7th.—No return of pains. Eye feels quite well. Scarcely any pain on right eye, and none on left. *The usual September aggravation has not occurred.*

